# Supplementary material for: Impact of work-family balance results on employee work engagement within the organization: The case of Slovenia
Source: PLoS One. 2021 Jan 20;16(1):e0245078. doi: 10.1371/journal.pone.0245078 (PMC7816978; doi:10.1371/journal.pone.0245078)
Supplement: S1 File — (DOCX) [file pone.0245078.s002.docx]

| **Demographic characteristics** | **Mean** | **SD** | **Med.** |
| --- | --- | --- | --- |
| Gender | 1.48 | .500 | 1.00 |
| Completed education | 3.42 | .787 | 3.00 |
| Size of the company | 1.70 | .827 | 1.00 |
| Job position | 2.27 | .777 | 2.00 |
| Working hour/week | 40.8 | 5.09 | 40.0 |
| Complexity of work | 2.12 | .765 | 2.00 |
| Marital status | 1.92 | .564 | 2.00 |
| Children | 1.28 | .448 | 1.00 |

| **Leader Support** | **Mean** | **SD** | **Med** |
| --- | --- | --- | --- |
| Switched schedules ( hours, overtime hours, vacation ) to accommodate my family responsibilities | 3.31 | 1.388 | 4.00 |
| Listened to my problems. | 3.67 | 1.020 | 4.00 |
| Takes into account my efforts to combine work and family | 3.60 | 1.027 | 4.00 |
| Juggled tasks or duties to accommodate my family responsibilities. | 3.51 | 1.100 | 4.00 |
| Shared ideas or advice. | 3.40 | 1.190 | 3.00 |
| Did not held my family responsibilities against me. | 3.16 | 1.478 | 3.00 |
| Helped me to figure out how to solve a problem. | 3.52 | 1.350 | 4.00 |
| Was understanding. | 3.80 | 1.122 | 4.00 |
| Did not showed resentment of my needs as a working parent. | 3.57 | 1.275 | 4.00 |
| **Co-worker support** | **Mean** | **SD** | **Med.** |
| Helped me to switch schedules ( hours, overtime hours, vacation ) to accommodate my family responsibilities | 3.73 | 1.192 | 4.00 |
| Listened to my problems. | 3.80 | 1.001 | 4.00 |
| Takes into account my efforts to combine work and family | 3.81 | 1.022 | 4.00 |
| Juggled tasks or duties to accommodate my family responsibilities. | 3.59 | 1.075 | 4.00 |
| Shared ideas or advice. | 3.63 | 1.076 | 4.00 |
| Did not held my family responsibilities against me. | 3.37 | 1.395 | 4.00 |
| Helped me to figure out how to solve a problem. | 3.63 | 1.289 | 4.00 |
| Was understanding. | 3.91 | 1.072 | 4.00 |
| Did not showed resentment of my needs as a working parent. | 3.62 | 1.286 | 4.00 |
| **Family-friendly policies and practices** | **Mean** | **SD** | **Med.** |
| Flexible working hours. | 3.29 | 1,439 | 4,00 |
| Flexible arrival / departure time. | 3.34 | 1,499 | 4,00 |
| Possibility of shortened working time (eg half-time). | 3.08 | 1,478 | 3,00 |
| Independence in the organization of replacements and on-call time. | 3.45 | 1,363 | 4,00 |
| Independence in the planning of annual leave. | 3.58 | 1,346 | 4,00 |
| Flexible working breaks (eg lunch time). | 3.76 | 1,260 | 4,00 |
| Children's bonus time (extra hours or extra leave for parents on the first day of the school, kindergarten introduction). | 3.31 | 1,427 | 4,00 |
| Possibility of working at a distance / from home. | 2.76 | 1,591 | 3,00 |
| Good communication with employees. | 3.01 | 1,506 | 3,00 |
| Research among employees regarding work-family balance. | 2.94 | 1,342 | 3,00 |
| A team for the work-family coordination, or an authorized person for issues related to work-family balance. | 2.44 | 1,303 | 2,00 |
| Informal socializing among employees. | 3,04 | 1,323 | 3,00 |
| Leadership supporting work-life balance measures and policies. | 3,08 | 1,262 | 3,00 |
| Leader is educated in the field of work-family balance. | 2,77 | 1,251 | 3,00 |
| Leadership support and promote the work.family balance. | 2,77 | 1,295 | 3,00 |
| Help to reintegrate into work after a long absence (for example, holiday leave). | 3,12 | 1,247 | 3,00 |
| Individual career development plans. | 3,01 | 1,270 | 3,00 |
| Annual interviews that include the topic of work-family balance. | 2,78 | 1,290 | 3,00 |
| Giving gifts to newborns or children at Christmas / New Year. | 3,27 | 1,412 | 3,00 |
| Leisure offer (the company organizes activities that can be used by employees or their family members). | 3,22 | 1,437 | 3,00 |
| Scholarships for employees' children. | 2,52 | 1,424 | 3,00 |
| Psychological counseling and help. | 2,27 | 1,345 | 2,00 |
| Various forms of daily care (for the children of their employees). | 2,06 | 1,333 | 1,00 |
| Organized holiday care for the children of their employees. | 1,83 | 1,223 | 1,00 |
| Possibility to bring children to a special situation for a short time to work. | 2,12 | 1,403 | 1,00 |
| **Work-family balance** | **Mean** | **SD** | **Med.** |
| The current relationship between the time I spend on the job and the time I have for my non-formal activities seems good to me. | 2.96 | 1.429 | 3.00 |
| I have problems with balancing work and non- work activities. | 3.18 | 1.094 | 3.25 |
| I think that the balance between my work requirements and non-work activities is just right. | 3.23 | 1.239 | 3.00 |
| Generally speaking, I think my work and private life is balanced.. | 3.25 | 1.212 | 3.00 |
| **Work engagement** | **Mean** | **SD** | **Med.** |
| At my work, I feel bursting with energy | 3.28 | 1.198 | 3.00 |
| At my job, I feel strong and vigorous | 3.88 | .850 | 4.00 |
| When I get up in the morning, I feel like going to work | 3.39 | 1.099 | 4.00 |
| I am enthusiastic about my job | 3.28 | 1.036 | 3.00 |
| I am proud on the work that I do | 3.61 | 1.019 | 4.00 |
| My job inspires me | 3.41 | 1.072 | 3.00 |
| I am immersed in my work | 3.42 | 1.011 | 3.00 |
| I get carried away when I’m working | 3.35 | 1.045 | 3.00 |
| I feel happy when I am working intensely | 3.48 | 1.105 | 4.00 |
