# Supplementary material for: Impact of work-family balance results on employee work engagement within the organization: The case of Slovenia
Source: PLoS One. 2021 Jan 20;16(1):e0245078. doi: 10.1371/journal.pone.0245078 (PMC7816978; doi:10.1371/journal.pone.0245078)
Supplement: Attachment — Submitted filename: Response to Reviewers.docx [file pone.0245078.s003.docx]

REBUTTAL LETTER

**A manuscript entitled »Impact of work-family balance results on employee work engagement within the organization: The case of Slovenia«**

Dear editors and reviewers,

We thank the reviewers for their careful reading of the manuscript and their constructive remarks. All the comments were carefully considered and taken into account. The paper has been revised accordingly. Please find in the table below a detailed point-by-point response to all comments.

Best wishes,

Authors

| **ID** | **Reviewers' comments** | **Response and revisions by authors** |
| --- | --- | --- |
|  | **Reviewer #1** | |
| R1.0 | The authors are interested in the relationship between work-family balance (WFB) and work engagement (WE), through the lens of organizational factors that might affect both. This is an important question, and I am glad the authors are researching it.  Generally, the paper is a simplistic analysis of the relationship between work-family balance and work engagement, without any actual test of that relationship. Nevertheless, there is some benefit to this paper, and the data seem particularly interesting (despite the low response rate).  There are some fundamental issues that the authors need to address prior to publication. | The authors would like to thank the reviewer for the comment |
| R1.1 | First, their definitions of their main dependent variables (DV), WFB and WE are missing entirely (although it is possible they intend for the reader to use the Andrew & Sofian definition of WE, it’s unclear). Though they do discuss how some other researchers have understood those terms, the reader is not clear how the authors do. Those definitions need to be clear.  The variables also need to be clearly described in their Methods section. Currently, WE is not mentioned at all, and WFB has no examples of the types of measures that are included in their 4-item scale. | To address this comment, we have included a definition of work-family balance and work engagement. The text is pasted below.  *»If we summarize the individual definitions of work-family balance, the concept can be described as satisfactory inclusion or adjustment between two roles in an individual’s life, namely work and family (Bedarkar & Pandita, 2014). The concept of work-family balance is mainly used to describe the stability and balance between responsibilities related to work and family, and the balance is where the individual believes that it is right (Rajni & Ravinder, 2015). Work-family balance is therefore understood as the effort of an individual to have enough time and energy to devote to the family, while performing all the tasks in his workplace.«*  *»The work engagement is defined as a psychological, positive, perfecting, work-related state characterized by vitality, dedication and absorption (Schaufeli & Bakker, 2004; Schaufeli, Salanova, Gonzalez-Roma & Bakker, 2002). Vitality means that the individual has a high level of energy during work and is mentally resilient. Dedication refers to the fact that the employee is strongly involved in their work and at the same time experiences a sense of importance, enthusiasm and challenge. Absorption means that the individual is completely immersed in his work, his time at work is running fast and he is hardly mistaken at work (Schaufeli & Bakker, 2003; Schaufeli & Bakker, 2004). Work engagement is therefore a relatively permanent state of mind that refers to the simultaneous investment of personal energy in work experience or success (Christian, Garza & Slaughter, 2011).«*  We have also included examples of the types of measures for work-family balance and describe measures for work engagement. The text is pasted below.  *»Respondents rated the statements regarding their work-family balance such as »The current relationship between the time I spend on work and the time I have for family activities seems good to me« or »Overall, I think my work and family life is balanced.«*  *»The last set of questions measures the construct of work engagement. To determine the individual's work engagement we used the Utrecht work engagement scale (Schaufeli & Bakker, 2003). We used a shorter version consisting of nine statements, three for each dimension of engagement. According to the Utrecht work engagement scale, engagement consists of three parts: vigor, dedication and absorption. Respondents indicated on a 5-point scale (1 - I do not agree at all, 5 - I completely agree) the extent to which they agree with the statements describing their feeling in the workplace (e.g. »I am full of energy at work« or »I am happy when I work intensively«).«* |
| R1.2 | Second, the authors mention both WFB and work-life balance in various places in their paper. I am aware that some researchers have used these phrases interchangeably, but others have carefully denoted the difference. It would be good if the authors clarify their own position on this. Providing the definition will help. | To address this comment, we have included a new paragraph which explains how the terms work-life balance and work-family balance differ. We also added definitions. The text is pasted below.  *»There are two terms in the literature to define a concept that describes the search for a balance between work and other life roles, namely work-life balance and work-family balance (Joseph, Jose Sebastian, 2019). Work-life balance is a concept that supports an individual’s effort to divide their time and energy between work and other important roles and tasks in their lives such as family, friends, community, spirituality, personal growth, hobbies and other private activities (Praphu Shankar , Mahesh, Nanjundeswaraswamy (2007). On the other hand, Work-family balance is the accomplishment of role-related expectations that are negotiated and shared between an individual and his or her role-related partners in the work and family domain (Grzywacz, Carlson, 2007). The terms are similar but not identical because the work-life balance construct is more comprehensive and broader than the work-family balance construct (Joseph, Jose Sebastian, 2019). In this paper, we use the term work-family balance, as we only examine the search for work-family balance.«*  In the text, we also unified the naming and used the term work-family balance everywhere |
| R1.3 | Third, the authors make a point of saying at the beginning the importance of a gendered analysis in this substantive area, but they do not complete one in this paper. That is a glaring absence. Given their sample is balanced by sex, I would encourage them to minimally provide some basic analyses of overall scores on all of their independent and dependent variables by women & men. (I believe the other demographics can wait for a later paper.) Of course including gender in their regressions is ideal, along with interaction variables, but minimally creating some reader knowledge about gender differences is important. | To address this comment we have provided basic analyses of overall scores on all independent and dependent variables by women and men. For all variables, we also performed T-tests to determine whether gender differences were statistically significant. The results of basic analyzes and analyzes of T-tests were briefly presented. The text is pasted below.  *»Our sample was balanced by gender, as the sample consisted of 178 men and 165 women. Gender plays an important role in work-family balance, so we found out how the answers for each variable differ by gender. To examine whether there are statistically significant differences between the gender in the perception of elements of organization, work-family balance and work engagement, we used the T-test. The results of the T-test showed statistically significant differences between the sexes only for the variables work complexity and work-engagement (p <0.5). Men thus rated their work as more coplex than women, as the average number of responses on the complexity of their work was 1.9 and for women 2.35 (1 = very complex, 5 = very uncomplex). In terms of work engagement, the answers showed that women are statistically significantly more engaged at work than men. The average for men was 3.38 and for women 3.54.*  *For the other variables, the differences by gender were not statistically significant, but the values still differed slightly between the gender. Men worked an average of 40.9 hours per week and women 40.8 hours. Leaders' support was rated almost equally by both sexes, with an average of 3.51 for men and 3.50 for women. There was a slightly larger difference in the support of co-workers, where the average for men was 3.61 and for women 3.74, which means that women rated their co-workers' support in work-family balance slightly better. Women also rated the presence of family-friendly policies and practices in their company better, as the average for women was 3.03 and the average for men was 2.92. There were also minor gender differences in the assessment of work-family balance performance. Men rated work-family balance somewhat surprisingly worse than women, with the average being 3.14 for men and 3.23 for women.«* |
| R1.4 | This also speaks to a larger issue with the paper—the reader has no way to interpret the results on the DV because it is not clear how the sample scored on those variables. Generally, was the sample positive about organizational policies and practices? Leader support? Peer support? WFB or WE? Those basic analyses should be presented briefly so readers can ascertain the scale of the effects being discussed also. | To address this comment we have expanded the method section and included a new subsection (2.3) with basic analyzes for dependent and independent variables and briefly presented the results of these analyzes. The text is pasted below.  *»We calculated mean values and standard deviations for individual questionnaires in relation to the variables included in our study. We were interested in how the sample generally assessed the variables under investigation. The results are presented in Table 1.*  *Respondents rated the complexity of their work on a 5-point scale from 1 (not complex at all) to 5 (very complex). As can be seen from Table 1, on average, respondents generally rated their work as complex. The average number of working hours is 40 hours per week. However, it should be noted that the standard deviation is quite high (5.1), as the respondents' working hours ranged from 8 to 80 hours per week. Nevertheless, most respondents answered that they worked the usual 40 hours per week. Respondents were also asked to assess how often an individual leader and his or her co-workers use certain supportive behaviors to balance an employee's work and family life (1 meant never and 5 meant always). In general, respondents rated both leader and co-workers' support as relatively high. The average co-worker support overall was rated slightly higher than leader support and the standard deviation was higher.*  *In connection with the company's family-friendly policies and practices, we asked the respondents what measures their company implements. Most present are measures in the area of work flexibility (e.g. flexible work breaks, flexible working hours, flexible arrival and departure times for work) and measures for self-control or independence (e.g. independence in planning annual leave and in organizing substitutions and on-call times). Least present are measures related to the protection of workers' children (e.g. various forms of day care for workers, organized holiday care for workers and the possibility of bringing their children to work part-time). Overall, respondents rated the representation of measures in their company as relatively poor, averaging 2.74 (1 means "strongly disagree", 5 "very agree").*  *We also calculated averages and standard deviations for the dependent variables, i.e. work-family balance and work engagement. The values for work-family balance are slightly above average, which means that the respondents are more satisfied than dissatisfied with work-family balance, but the answers of the respondents are quite different, as the standard deviation is 1.1. The respondents also rated their work engagement relatively high, with the dimension of vigor being highest and that of absorption lowest.«*  To clarify the results we also included a new Table (Table 1). |
| R1.5 | Also in terms of methodology, I am unclear why they are calling this a case study. They are not looking within an organization, but rather across organizations. That’s a cross-sectional design. It would also benefit the reader if we knew if their sample demographics (including percent working in each sector) is comparable to population statistics. I also needed clarification between administration and management, because those terms are often used interchangeably in the literature. | In subsection 2.1, we have changed the naming “case study” into “cross-sectional design” as suggested by the reviewer. We have added major population statistics to make it easier for the reader to imagine how comparable our sample is in terms of population. The text is provided below.  »*According to the Statistical Office of the Republic of Slovenia, in 2017 (the last available data in 2019, when the survey was conducted), 195,756 companies were registered in Slovenia, of which 185,997 micro-enterprises (0 to 9 employees) and 7,329 small enterprises 49 employees), 2,084 medium-sized enterprises (from 50 to 249 employees) and 346 large enterprises (over 250 employees) (STAT, 2019).«*  *»In Republic of Slovenia are current 968,000 persons in employment (STAT, 2020).«*  Despite the reviewer's suggestion, we did not include data on how many people in Slovenia work in leading positions, how many in administration and how many in production. We are aware of the fact that this information would offer the reader a broader insight into the representativeness of the sample by sector, but unfortunately we did not find data in slovenian databases.  To make it easier to understand and to avoid confusing these two concepts, we have clarified between administration and management and explained these two concepts in subchapter 2.2. The text is passed below.  *"In the research, we have divided positions or jobs into three groups, ie management, administration and production. Each of the three groups has its own specifics important for the work-family balance (eg responsibility, the possibility of flexible place and time of work, the complexity of work and the like). Management in our case means lower, middle and top management or leaders whose task is management and leadership; administration means all support and operational services that are important to the functioning of the organization and are not management or production (for example, finance, marketing, human resources and others)."* |
| R1.6 | In the Results, complexity of the work is said to be negatively associated with WFB, but the table shows positive. That needs clarification. Table 3 can also be more easily interpreted if we understand how WE is measured. | We thank the reviewers for a detailed review of the paper and comment. As it turns out, we included the wrong values in the regression table. We corrected the error and performed a regression analysis again and found that the result in the calculation was negative, ie - 0.165. Other values also changed slightly, so we corrected the entire table of regression values. The table (extract from SPSS) is attached below. We thank the reviewers again for the warning. **Coefficients^a^** \| \| \| \| \| \|; Model \| \| Unstandardized Coefficients \| \| Standardized Coefficients \| t \| Sig.; \| \| B \| Std. Error \| Beta \| \|; 1 \| (Constant) \| 2,676 \| ,546 \| \| 4,901 \| ,000; \| zahtevnostOBR \| -,165 \| ,071 \| -,115 \| -2,325 \| ,021; \| število delovnih ur na teden \| -,007 \| ,011 \| -,033 \| -,663 \| ,508; \| ukrepi \| ,523 \| ,067 \| ,387 \| 7,803 \| ,000; a. Dependent Variable: WLB \| \| \| \| \| \| We add explanation about work engagement measures in sub-section 2.1 (Instrument). See answer on comment R1.1 |
| R1.7 | They also misspoke in the first and 2nd para after Table 3. H3 requires WE to be DV. If they did a multilevel regression with WFB as a mediating variable between organizational factors and WE, it should have been analyzed and presented as such. Table 4 does not look like that at all. So, the write-up and the table need to be better aligned to clarify the actual analysis conducted. This is especially important because on the next page the authors say: “A work-family balance makes an important contribution to work engagement, as research showed that those who had a more balanced work and family life were more engaged in the workplace.” The analyses shown do not verify that conclusion, as WFB and WE are not in the same analysis anywhere in the paper. | We thank the reviewer for that comment. We did not identify mediation in the analysis because we considered the impact of one concept on another separately and because the results confirmed the connection between the discussed concepts, we did not find it meaningful to identify WFB as mediatior. More specifically, we used regression analyzes to determine the impact of elements on the organization's level on the WFB, the impact of the WFB on the WE, and ethe organization's lements that support the WFB (or more specifically an individual's perception of organizational support) on the WE. For greater clarity, we have supplemented the model and presented it in a more understandable way.  The sentence “A work-family balance makes an important contribution to work engagement, as research has shown that those who had a more balanced work and family life were more engaged in the workplace.” refers to the results associated with Hypothesis 2, which is »Work-family balance influence the work engagement«. The results of the regression analysis showed that WFB has a statistically significant effect on WE, which is shown in Table 3. Under Hypothesis 2, there is also a detailed interpretation of the statistical results. For better transparency, the results of statistical analyzes and interpretation of results were divided into 3 subchapters according to each hypothesis. |
| R1.8 | The last sentence prior to the conclusion also goes beyond their reported results by suggesting again a relationship between FWB and WE- in this case that the absence of FWB can be related to WE because of organizational factors. I see no evidence of this analysis in the paper. | As the reviewer noted the last sentence before the conclusion *(“At the same time, those who do not have enough balance in both areas can also get involved in their work, which is encouraged by the fact that they feel that the organization helps them to find a balance.»)* is not sufficiently supported by the results, so we deleted this claim from the text. |
| R1.9 | Finally, there are some writing mistakes in the paper, such as misspellings or missing/extra words, but the paper is generally well-written. | The authors would like to thank the reviewer for the comment. We did the grammary check. |
|  | **Reviewer #2** | |
| R.2.0 | Very interesting paper, but I have some suggestions for your considerations. | The authors would like to thank the reviewer for the comment. |
| R2.1 | The key words should be revised, "Work-family balance" and "work engegement" should be fine, but "model" maybe is not a good term here, you can consider some other words, like "organization management"? | We have revised key words. We have removed term “model” and replaced it with word “organization management” as reviewer suggested |
| R2.2 | More details should be added for proposing the three hypotheses, and it should be taken as an idenpendent section. | To address this comment we have added new sub-section (1.1) and added more details for proposing our hypotheses. Text is passed below.  *»1.1 Hypotheses*  *On the basis of the above, we have formulated three hypotheses to determine the relationship between organizational support for work-family balance, work-family balance and job engagement. We propose that organizational support for work-family balance has a positive effect on the individual's work-family balance, that work-family balance has a positive effect on the individual's work engagement and that the perception of organizational support for work-family balance has a positive effect on the individual's work engagement.*  *Numerous studies underline the importance of support in promoting a working environment that helps employees to balance work and family life (e.g. Greenhous, Ziegert & Allen, 2012; Thomas & Ganster, 1995; Trefalt, Drnovšek, Svetina-Nabergoj & Adlešič, 2013). Most research emphasizes that organizations can support the work-family balance of their employees through family-friendly policies and practices that help employees balance work and family responsibilities (Boyar, Maertz Jr & Mosley, 2008). In any case, as Knaflič, Svetina Nabergoj and Pahor (2010) emphasize, it is important that organizations implement a sufficient number of policies and that these are adapted to the organization and the needs of its employees. Research (Adkins & Premeaux, 2012; Bohle, Willaby,Quinlan & McNamara, 2011; Stier, Lewin-Epstein & Braunc, 2012) has shown that the length of working time also plays an important role in reconciling work and family life, as those employees who have a longer working day have greater difficulty in reconciling work and family life. Research (Bohle et al., 2011) has shown that in addition to the length of the working day, the complexity of the work also has a negative influence on the work- family balance. In particular, management has an influence on the creation of a working environment that supports the work- family balance and as studies have shown (Knaflič, Svetina Nabergoj & Pahor, 2010), management support is also the key to the successful implementation of family-friendly measures. Management support is important for the work-family balance in all career phases, especially at the beginning of the career and before retirement (Darcy, McCarthy, Hill & Grady, 2012). Study by Griggs, Casper and Eby (2013) has shown that, in addition to management, co-worker support also makes an important contribution to a stimulating work environment and the work- family balance. Based on previous research that emphasizes the importance of elements at the organizational level, such as the leader and co-workers support, family-friendly policies and practices, as well as the length of the working day and the complexity of the work, we propose hypothesis 1.*  *H1: Elements at the organizational level influence the work-family balance of the individual.*  *A study examining the direct impact of the work-life balance of an individual on his work engagement has not yet been conducted, but the results of the Kar & Misra (2013) survey confirmed that those employees who receive employer support in coordinating work and family are more satisfied at work and more engaged. At the same time, research has also shown that family-friendly organizational measures influence an individual’s greater work engagement (Caesens & Stinglhamber, 2014; Mache, Vitzthum, Klapp & Danzer, 2014; Taghipour & Dezfuli, 2013). Based on this previous knowledge we formulated Hypothesis 2.*  *H2: Work-family balance influence the work engagement.*  *Research (Valcour, Ollier-Malaterrein, Matz-Costa, Pitt-Catsouphes & Brown, 2011) confirmed that job requirements and family-friendly policies and practices shape individuals' perceptions of organizational support in work-family balance. This happens through two mechanisms: on the one hand, the organization shows that it cares about the balance or work-life balance of employees and, on the other hand, it helps them to develop and maintain ways to meet the needs and demands of work and daily life. A study by Swanberg, Ojha, Mckechnie and Boone James (2011) found that some family-friendly measures, such as supporting managers and giving employees control over their schedules, not only help to balance work and family, but also have a significant impact on increasing employee engagement. Work engagement is also influenced by support of work organization (Caesens & Stinglhamber, 2014) and support of co-workers and the social environment (Mache, Vitzthum, Klapp & Danzer, 2014) or, as defined by Taghipour and Dezfuli (2013), by the moral climate in the organization. Based on this previous knowledge we formulated hypothesis 2.*  *H3: Individuals' perception of the organization's support for work-family balance influences their work engagement.*  *The proposed hypotheses were tested in the proposed model (Figure 1) as follows.«* |
| R2.3 | I suggest to divide the section 3 into 3 sub-sections by corresponding to the three thypotheses. | As suggested by reviewer we have devided the section 3 into 3 subsection (3.1 Hypothesis 1 - Elements at the organizational level influence the work-family balance of the individual.; 3.2 Hypothesis - Work-family balance influence the work engagement; 3.3 Hypothesis 3 - Individuals' perception of the organization's support for work-family balance influences their work engagement.) by corresponding to the 3 hypotheses |
