# Supplementary material for: Impact of work-family balance results on employee work engagement within the organization: The case of Slovenia
Source: PLoS One. 2021 Jan 20;16(1):e0245078. doi: 10.1371/journal.pone.0245078 (PMC7816978; doi:10.1371/journal.pone.0245078)
Supplement: Attachment — Submitted filename: Response to Reviewers2.docx [file pone.0245078.s004.docx]

REBUTTAL LETTER

**A manuscript entitled »Impact of work-family balance results on employee work engagement within the organization: The case of Slovenia«**

Dear editors and reviewers,

We thank the reviewers for their careful reading of the manuscript and their constructive remarks. All the comments were carefully considered and taken into account. The paper has been revised accordingly. Please find in the table below a detailed point-by-point response to all comments.

Best wishes,

Authors

| **ID** | **Reviewers' comments** | **Response and revisions by authors** |
| --- | --- | --- |
|  | **Reviewer #1** | |
| R1.0 | The authors have substantially rewritten their paper, clarifying much of their terminology and analyses. Their results are now clear. The conclusions they reached in their prior paper have now been largely confirmed here. There is a bit of redundancy added into the literature review now, but it does not substantially detract from the paper's arguments. | The authors would like to thank the reviewer for the comment |
| R1.1 | Their writing, however, continues to have problems and will require careful editing prior to publication. These mistakes are minimal, but frequent. | We reviewed and edited the article. The article was also edited by an official english proofreader. |
| R1.2 | One mistake remains in the paper- on p 19 the authors state that men's jobs are more complex. However, they reverse the codes for complexity to make this argument (found 3 other places in the document coded differently). I find it ironic that they invoked a masculine understanding of complexity to give men more complex jobs (by misinterpreting their own coding) when their own data suggest that's not the case. | There was a writing error. We have corrected the data. We thank the reviewers for a detailed review and exposure of nonconformities. The correction can be seen from the text. |
| R1.3 | Finally, in the new sections added the authors have used solely male pronouns. Given that the rest of the document employes a more gender neutral approach, I recommend they continue with that neutral approach and revise the new sections accordingly. | We corrected solely male pronouns where it was noticeable and didn’t make sense given the context. The article was also edited by an official english proofreader. |
|  | **Reviewer #2** | |
| R.2.0 | I would like to suggest that this manuscript should be considered for publication in the journal PLOS one | The authors would like to thank the reviewer for the comment. |
